# Item Difficulty of Fugl-Meyer Assessment for Upper Extremity in Persons With Chronic Stroke With Moderate-to-Severe Upper Limb Impairment

**DOI:** 10.3389/fneur.2020.577855

**Published:** 2020-11-16

**Authors:** Nanako Hijikata, Michiyuki Kawakami, Ryota Ishii, Keita Tsuzuki, Takuya Nakamura, Kohei Okuyama, Meigen Liu

**Affiliations:** ^1^Department of Rehabilitation Medicine, Keio University School of Medicine, Tokyo, Japan; ^2^Biostatistics Unit, Clinical and Translational Research Center, Keio University Hospital, Tokyo, Japan

**Keywords:** stroke, hemiparesis, assessment, Rasch, upper limb

## Abstract

**Background and Purpose:** Limited research has been conducted with the aim of understanding which upper extremity movements are difficult for persons with severe chronic stroke. The purpose of this study was to test the structure of the Fugl-Meyer Assessment for Upper Extremity (FMA-UE) using Rasch analysis in persons with chronic stroke with moderate to severe deficits and to determine the item difficulty hierarchy.

**Methods:** This was a secondary analysis of data from previous randomized, controlled trials, or clinical trials. The participants were 101 persons with chronic stroke with moderate to severe hemiparesis (time after onset of stroke, 1375.3 ± 1157.9 days; the 33-item FMA-UE, 31.1 ± 12.8). Principal component analysis and infit statistics were used to evaluate dimensionality. Rasch analysis using a rating scale model was performed, and item difficulty was determined.

**Results:** Six misfit items were removed. The results showed that the 27-item FMA-UE was unidimensional. Rasch analysis showed that the movements performed within synergies were among the easiest items. Shoulder and elbow movements were among the easiest items, whereas forearm and wrist movements were among the moderately to most difficult items. Hand items spanned various difficulty levels.

**Discussion and Conclusions:** The FMA-UE is a valid assessment tool of upper extremity motor function in persons with chronic stroke with moderate to severe deficits. The results showed that item difficulty was consistent with the stepwise recovery course proposed by Fugl-Meyer. The movements that are difficult for patients with moderate to severe chronic paresis were determined, which would enable comparison of each movement using a measure of motion difficulty in future studies.

## Introduction

With advances in technology, severe chronic motor impairment of the upper limbs is now one of the major targets in stroke rehabilitation (1). Although motor recovery used to be thought to reach a plateau chronically in persons post-stroke, substantial motor improvement was shown after repetitive task training or constraint-induced movement therapy (2–5). These training regimens have been developed based on the theory of motor learning (6–8), defined as the repetition-mediated increase in the speed and accuracy of a newly acquired motor behavior (9). With repetition of the selected behaviors, the highly stereotyped motor skill is finally acquired, and this process results in an expansion of neuron ensembles in the motor cortex (10). Technology-aided interventions, such as robotics and neuromuscular electrical stimulation, offer the opportunity of repetitive motor training for persons post-stroke with severe motor deficits. However, conflicting reports (1, 4, 11) shows lack of consensus regarding the effectiveness of proximal vs. distal or mono- vs. multi-joint approaches. The lack of studies about key movements predicting motor recovery or response to interventions might contribute to this. Importantly, no studies have evaluated which upper extremity movements are difficult to perform for persons with severe chronic stroke. Clarification of these issues would facilitate the decision of where to set priorities in planning rehabilitation strategies.

The Fugl-Meyer Assessment (12) is the gold standard to assess motor function of post-stroke hemiparesis (13, 14). The Fugl-Meyer Assessment for Upper Extremity (FMA-UE) has sound psychometric properties of reliability (15–20), validity (15–17, 20), and responsiveness (15, 16, 19, 20). Each item consists of movements reflecting motor function in post-stroke hemiparesis, spanning from proximal to distal joints. Determining the item difficulty of the FMA-UE is not only useful for an accurate evaluation of upper extremity paresis, but it is also applicable to rehabilitation practice. Woodbury et al. analyzed the structure of the FMA-UE using Rasch analysis and reported the item difficulty hierarchy (21, 22). However, the majority of the target population were persons with acute stroke with mild to moderate motor deficits. It is therefore necessary to test whether the item difficulty hierarchy is the same in persons with severe chronic stroke. Accordingly, the purpose of this study was to test the structure of the FMA-UE using Rasch analysis in persons with chronic stroke with moderate to severe upper extremity motor deficits, and to determine the item difficulty hierarchy.

## Materials and Methods

### Study Design

This study was a secondary analysis of data from previous randomized, controlled trials or clinical trials (23–26). Outlines of each study, including ethical approval and clinical trial registration numbers, are provided as Supplementary Material. In this study, all persons who had participated in these trials at Keio University Hospital between April 2017 and June 2019 were included. For participants who were hospitalized several times during the study period, the assessment implemented on the first admission was used, excluding the data from the second and subsequent admissions. One of the authors, blinded to the interventions, extracted the data from the medical records of the participants.

This study was approved by the institutional ethics review board (20190144). The outline of the study was published on the public website, and the participants were guaranteed the right to refuse participation.

### FMA-UE

The FMA-UE was used as an outcome measure in the clinical trials. The FMA-UE consists of 30 items assessing motor function and 3 items assessing reflex function. The score most applicable to task performance is given from “0, inability,” “1, beginning ability,” to “2, normal” (total score range, 0–66). Based on the standardized guideline developed by Platz et al. (27) the FMA-UE was administered by trained physiatrists before and after the treatment/intervention. This study used the pre-treatment/intervention data. The assessors were trained as follows: they were instructed to (a) review the standardized guideline developed by Platz et al. (27); (b) watch the training video developed by See et al. (19) (ArmFM_TrainingVideo); (c) watch the subject 1 video (ArmFM_TestSubject1) (19) and score the patient; (d) review the answer (ArmFM_AnswerKey_Subject1) (19); (e) repeat processes (c) and (d) for subject 2; (f) watch the assessment by an attending physiatrist with more than 10 years of experience and score the patient at the same time; (g) review the two scores and note scoring discrepancies; (h) repeat processes (f) and (g) until the score discrepancies become <2, set below the MDC (3.2 points) (19) under which values could be regarded as measurement error); and (i) assess the patient using the FMA-UE and get feedback from the attending physiatrist (at least 3 times).

### Participants

The participants were 101 persons with chronic stroke (time after onset of stroke, 1375.3 ± 1157.9 days). The participants' demographic characteristics are presented in Table 1. The Stroke Impairment Assessment Set (28) was used to assess motor and sensory impairment in the affected upper limb. The modified Ashworth scale (29) was used as a measure of resistance to passive movement. The severity of motor impairment for a paretic upper limb was evaluated using the 33-item FMA-UE. In this study, FMA-UE >45 was defined as mild, ≥30 and ≤ 45 was defined as moderate, and <30 was defined as severe, according to the previous study (1). Most of the participants (85%) were classified as moderate or severe, whereas a small number (15%) were classified as mild. Similar proportions of persons with different severities were included in a previous study (21), and therefore, it was decided to include mildly affected persons in the subsequent analysis.

**Table 1:** Demographic characteristics of the participants.

	***N =* 101**
Age (y)	54.8 ± 13.5^a^
Sex	
Males	62 (61.4)^b^
Females	39 (38.6)^b^
Time since stroke (days)	1375.3 ± 1157.9^a^
Range (days)	201–6,202
Affected side	
Right	59 (58.4)^b^
Left	42 (41.6)^b^
Is affected side dominant?	
Yes	44 (43.6)^b^
No	57 (56.4)^b^
Stroke type	
Ischemic	42 (41.6)^b^
Hemorrhagic	57 (56.4)^b^
Other	2 (2.0)^b^
Stroke location	
Right hemisphere	43 (42.6)^b^
Left hemisphere	56 (55.4)^b^
Brainstem	2 (2.0)^b^
33-item FMA-UE	29 [21–38]^c^
Severity^*^	
Severe	53 (52.5)^b^
Moderate	32 (31.7)^b^
Mild	16 (15.8)^b^
Stroke impairment assessment set	
Knee-mouth	4, *n =* 11; 3, *n =* 74; 2, *n =* 16
Finger	≥2, *n =* 15; 1c, *n =* 20; 1b, *n =* 12; 1a, *n =* 54
Light touch	3, *n =* 46; 2, *n =* 27; 1, *n =* 23; 0, *n =* 3
Position sense	3, *n =* 62; 2, *n =* 5; 1, *n =* 22; 0, *n =* 10
Modified ashworth scale^**^	1 (1,2)^c^
Elbow	≥1, *n =* 76 (75.2)^b^
Wrist	≥1, *n =* 79 (78.2)^b^

* The severity was defined using the 33-item FMA-UE (range, 0–66): severe, <30; moderate, ≥30, ≤ 45; and mild, >45.

** Score 1+ was transformed to 2, and scores 2 and 3 were transformed to 3 and 4.

a mean ± SD,

b n (%),

c *median [interquartile range]*.

Participants received hybrid assistive neuromuscular dynamic stimulation (HANDS) therapy (23) or participated in other randomized, controlled trials or clinical trials (24–26). The common characteristics of the population were as follows: the time from stroke onset was longer than 180 days; participants had the ability to walk independently with/without a cane and/or an orthosis; and participants experienced no significant pain or apparent contracture on the paretic upper limb. Detailed inclusion/exclusion criteria are presented in the Supplementary Material.

### Statistical Analyses

#### Score Distribution and Local Independence

Before performing Rasch analysis, the distribution of each item score was overviewed and items for which all participants had the same scores were removed. Subsequently, the items were screened to determine whether they would violate the two assumptions of Rasch analysis: local independence and unidimensionality. Local independence means that an item being measured is independent of the performance (and score) of any other item. That is, a certain performance in one item should never lead to any other item score. Unidimensionality means that each item for a measurement scale measures only one construct, that is, motor function of a paretic upper extremity in this context.

#### Dimensionality

To evaluate dimensionality, principal component analysis (PCA) and infit statistics were used. These statistical methods are commonly used to test the dimensionality of upper extremity outcome measures (30). For PCA, if a measurement scale could measure only one construct, in this case, upper extremity motor function, then the variance (i.e., eigenvalue) explained by the first factor would be very large. In the present study, factors with eigenvalues > 1 were extracted. The percent of total variance accounted for by the first factor was assumed to be 20–40% (31). In the present study, 40% was considered acceptable. Factor loadings, the extent to which each item is related to (i.e., loads on) the factors, were determined.

Fit statistics, calculated with Rasch analysis, are one of the most common indicators for testing the degree to which an item deviates from the assumption of unidimensionality (31). The values obtained from fit statistics are mean squares (MnSq) of residuals, the difference between observed scores for an item and expected values predicted by the model. High MnSq values indicate that the item does not fit the model. Infit statistics are less susceptible to outliers than outfit statistics. In the present study, infit MnSq values <1.7 were considered acceptable (31).

#### Rasch Analysis and Item Difficulty Hierarchy

After misfit items had been removed, another Rasch analysis was performed using a rating scale model (32). The rating scale model is an expanded model of the original dichotomous Rasch model and can be applied to measurement scales with multichromatic choices (e.g., 3-point scale of the FMA-UE) (30). This analysis to was used determine the item difficulty hierarchy, calibrated with logits.

R (R Foundation for Statistical Computing, Vienna, Austria) was used for the statistical analysis.

## Results

### Score Distribution and Local Independence

After the distribution of each item score had been overviewed, the following four items were omitted: “biceps reflex,” “triceps reflex,” “elbow flexion,” and “finger mass flexion.” For these items, all participants had the same or similar scores (that is, all participants obtained the highest score for the two reflex items; for the other items, the majority of participants had the highest score, and none of them scored zero), so these four items could not be dealt with in the rating scale model. In addition, the item “normal reflex” was removed because it was only assessed when the previous three items received the highest possible score, which thus interfered with local independence. For the other items, local independence was assumed to be maintained. Consequently, five items were removed before PCA and infit statistics.

### Dimensionality

The PCA identified five factors with eigenvalues > 1. The first factor accounted for 40.0% of the total variance, and the other four factors accounted for 13.4, 7.1, 4.9, and 3.8% of the variance, respectively. These results were then compared to those of previous studies (21, 22). and it was concluded that unidimensionality was preserved. The infit statistics revealed that the “hook grasp” item exceeded the acceptable range (Table 2). The infit statistics beyond the acceptable range made the item a candidate for removal, and the outfit statistics and factor loading were reviewed. This item showed abnormally high outfit statistics (outfit MnSq, 2.06; Table 2) (31). In addition, the factor loading value was not high (*r* = 0.46). These findings indicated that the “hook grasp” item was a misfit, so the item was removed from the subsequent analysis. With removal of this item, the Akaike Information Criterion decreased by 143.8, which indicated improvement of the goodness of fit. Finally, Rasch analysis of the 27-item FMA-UE was performed.

**Table 2:** Infit/outfit statistics of the 28-item Fugl Meyer Assessment for Upper Extremity^†^.

	**Infit MnSq**	**Outfit MnSq**
Scapular retraction	0.68	0.77
Scapular elevation	0.94	1.97
Shoulder abduction	0.70	1.95
Shoulder external rotation	1.06	1.43
Forearm supination	1.24	1.63
Shoulder adduction/internal rotation	0.75	1.13
Elbow extension	1.05	1.26
Forearm pronation	0.77	1.05
Hand to lumbar spine	0.65	0.56
Shoulder flexion 0–90°, elbow extended	0.91	0.81
Forearm pronation/supination, elbow at 90°	0.56	0.49
Shoulder abduction 0–90°, elbow extended	0.70	0.68
Shoulder flexion 90–180°, elbow extended	0.93	0.77
Forearm pronation/supination, elbow extended	0.49	0.40
Wrist stability, elbow at 90°	1.14	0.95
Wrist flexion/extension, elbow at 90°	0.68	0.55
Wrist stability, elbow extended	1.21	1.09
Wrist flexion/extension, elbow extended	0.74	0.57
Wrist circumduction	0.70	0.41
Finger mass extension	0.82	0.74
Grasp A, hook	1.77	2.06
Grasp B, thumb	1.19	1.71
Grasp C, pincer	1.17	1.15
Grasp D, cylindrical	1.48	1.38
Grasp E, spherical	0.85	0.75
Tremor	1.09	0.87
Dysmetria	1.25	0.94
Speed	0.99	0.64

MnSq, mean squares of residuals, represents the difference between observed scores for an item and expected values predicted by the model.

† *Biceps/triceps reflex, elbow flexion, and finger mass flexion were removed because all participants scored 1 or 2. Normal reflex was removed for local independence*.

### Rasch Analysis and the Item Difficulty Hierarchy

The results of the Rasch analysis are shown in Table 3. The item difficulty measures, calibrated with logits, were adjusted (i.e., normalized) so that the mean was 0 and the standard deviation (SD) was 1. The error values were standard errors of the item difficulty measures obtained by dividing the raw error values by the SD of the item difficulty measures in the original scale.

**Table 3:** Rasch item difficulty and infit/outfit statistics of the 27-item Fugl-Meyer Assessment for Upper Extremity^†^.

	Measure^*^	**Error**	**Infit MnSq**	**Outfit MnSq**
Shoulder adduction /internal rotation	−1.84	0.15	0.77	1.15
Shoulder abduction	−1.66	0.14	0.72	1.98
Scapular retraction	−1.66	0.14	0.67	0.73
Scapular elevation	−1.46	0.13	0.95	1.98
Elbow extension	−1.43	0.13	1.08	1.27
Shoulder external rotation	−1.00	0.11	1.06	1.44
Grasp D, cylindrical	−0.71	0.11	1.54	1.46
Shoulder flexion 0–90°, elbow extended	−0.59	0.11	0.92	0.82
Hand to lumbar spine	−0.44	0.11	0.66	0.57
Shoulder abduction 0–90°, elbow extended	−0.13	0.11	0.72	0.69
Wrist stability, elbow extended	0.02	0.11	1.24	1.12
Wrist stability, elbow at 90°	0.13	0.11	1.19	0.98
Forearm supination	0.22	0.11	1.28	1.69
Finger mass extension	0.34	0.11	0.87	0.83
Grasp E, spherical	0.40	0.12	0.89	0.79
Forearm pronation	0.42	0.12	0.82	1.10
Shoulder flexion 90–180°, elbow extended	0.53	0.12	0.91	0.75
Wrist flexion/extension, elbow extended	0.55	0.12	0.76	0.58
Wrist flexion/extension, elbow at 90°	0.55	0.12	0.70	0.55
Grasp C, pincer	0.57	0.12	1.22	1.19
Forearm pronation/supination, elbow at 90°	0.60	0.12	0.58	0.51
Dysmetria	0.64	0.12	1.25	0.93
Forearm pronation/supination, elbow extended	0.67	0.12	0.50	0.40
Tremor	0.87	0.13	1.11	0.87
Grasp B, thumb	0.98	0.14	1.25	1.84
Wrist circumduction	1.26	0.15	0.75	0.45
Speed	2.16	0.23	1.04	0.68

Each item is arranged in order of the difficulty calibrated with logits, with higher logits representing higher item difficulty.

† The three reflexes, elbow flexion, finger mass flexion, and hook grasp were removed.

* *The item difficulty measures were based on the logit value indicating transition from inability to beginning ability; values were adjusted so that mean was 0 and standard deviation was 1*.

#### Synergies vs. Coordinated Voluntary Movements

The movements performed within flexor/extensor synergies were confined to the easiest items. In contrast, all coordination/speed items were at the most difficult levels. The next easiest item out of synergies was “shoulder flexion to 90° with elbow extended.”

#### Shoulder/Elbow/Forearm

For each joint movement, all the shoulder and elbow movements except for “shoulder flexion 90–180, elbow extended” were among the easiest items. Forearm movements were among the moderate difficult items, and alternating movements such as forearm pronation/supination were more difficult than stabilized movements.

#### Wrist

Wrist movements were among the moderate to most difficult items. The difficulty increased from stability, alternating movement, to circumduction, regardless of elbow position.

#### Hand

Finger movements spanned various difficulty levels. “Finger mass extension” was in the middle overall. The difficulty of each grasp increased from “cylindrical (the easiest),” “spherical,” “pincer,” to “thumb adduction (the most difficult).”

**Figure 1:**
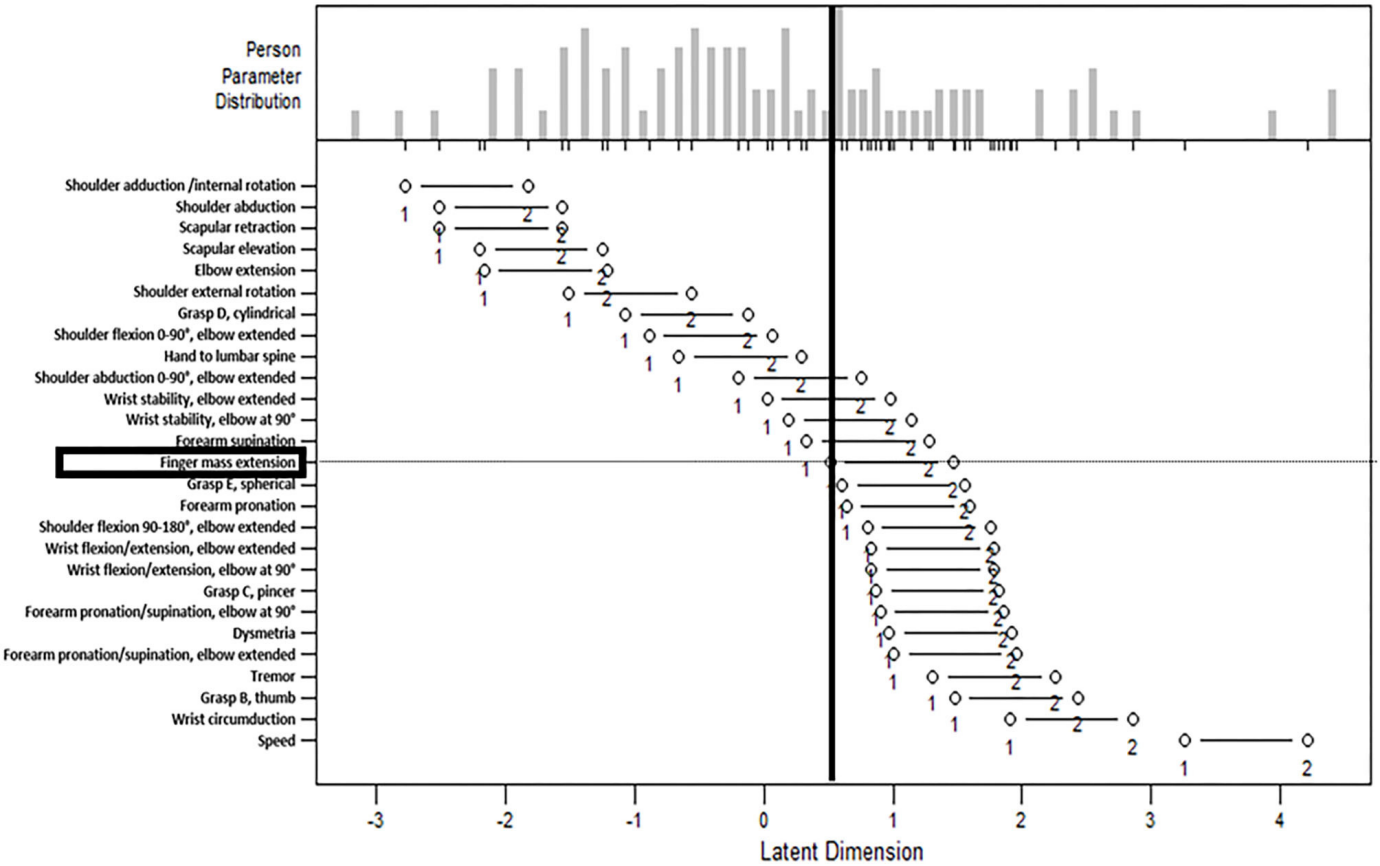
The person-item map. The person-item map with the person's ability plotted in the upper panel and the item difficulty plotted in the lower panel. The horizontal axis of the lower panel is a parameter of the item difficulty (calibrated with logits; values not normalized), with higher logits representing higher item difficulty. The horizontal axis of the upper panel is a parameter of the person's ability using the same scale as the parameter of item difficulty in the lower panel. The left white dot depicts the item difficulty measures. Item scores are likely to be 0 if a participant's ability is on the left side of the left white dot, 1 if a participant's ability is between two white dots, and 2 if a participant's ability is on the right side of the right white dot. The item “finger mass extension” is highlighted, and the vertical line divides the participants, which suggests that over half of the participants were incapable of extending their fingers.

The person-item map is presented in Figure 1. The person's ability is plotted as a histogram in the upper panel, and the item difficulty is plotted in the lower panel. The horizontal axis of the lower panel is a parameter of the item difficulty (calibrated with logits; values not normalized), with higher logits representing higher item difficulty. The horizontal axis of the upper panel is a parameter of the person's ability using the same scale as the parameter of item difficulty in the lower panel. The left white dot depicts the item difficulty measures, based on the logit value indicating transition from inability to beginning ability. Item scores are likely to be 0 if a participant's ability is on the left side of the left white dot, 1 if a participant's ability is between two white dots, and 2 if a participant's ability is on the right side of the right white dot. Figure 1 visualizes the distribution of persons capable of each upper extremity movement. For example, the item of “finger mass extension” is highlighted, and the vertical line divides the participants; this figure suggests that over half of the participants were incapable of extending their fingers.

## Discussion

In this study, the difficulty hierarchy of the FMA-UE was determined using Rasch analysis in persons with chronic stroke with moderate to severe upper extremity motor deficits. Rasch analysis has some advantages, such as the interval scale, item difficulty hierarchy, and unidimensionality (30). Rasch analysis enables determining the item difficulty hierarchy and comparing a person's ability with item difficulty using the equal interval scales, which can be helpful for setting appropriate rehabilitation goals targeted at the individual's ability. For example, Woodbury et al. generated the FMA-UE keyform recovery maps using Rasch analysis, thus translating a standardized measurement scale into a tool for designing treatment plans to provide optimally challenging tasks and progress task difficulty according to a person's ability (33). However, the item difficulty hierarchy of a measurement scale obtained using Rasch analysis changes across different target populations (30). The participants in this study had several different characteristics from those in the study by Woodbury et al. (21) one was the chronicity (time after stroke onset, 1375.3 ± 1157.9 vs. 16.9 ± 31.2 days), and another was the motor severity. This study, in which the 33-item FMA-UE was used, showed that the majority (85%) of the participants were persons with moderate to severe deficits. In contrast, Woodbury et al. (21) using the Orpington prognostic scale to define the severity of stroke, noted that the participants were predominantly persons with mild to moderate deficits, including only 10% with severe deficits. Although direct comparison is not possible because the definition of severity differs in the two studies, one finding suggests that the participants in the present study had more severe upper motor deficits than those in the previous study (21); the present study showed that over the half of the participants were incapable of finger extension, whereas the motion was easy for the participants in the study by Woodbury et al. (21). Thus, the present study was conducted on the assumption that the item difficulty hierarchy of the FMA-UE would differ from the previous study (22) in persons with chronic stroke with moderate to severe stroke.

### Dimensionality

Unidimensionality, which is one of the assumptions of Rasch analysis, can also indicate the structural validity of a measurement scale; that is, only one construct is measured, which in this case was upper limb motor function. The present PCA and infit statistics results showed that the 27-item FMA-UE was unidimensional. The PCA showed that the percent of variance explained by the first factor was relatively low compared with the results reported by Woodbury et al. (21). Post-stroke hemiparesis is associated with increased spasticity, increased stiffness (and reduced compliance) of muscles, soft tissue contracture, reduced muscle strength, and maladapted synergy formation over time (34–36). Furthermore, persons with more severe paresis are reported to have a higher risk of developing spasticity (37). In fact, the percentage of the participants with MAS ≥1 in this study was higher than in the previous report by Wissel et al. (38). Similarly, abnormal upper limb synergy and compensatory movements are likely to be observed in moderately to severely impaired persons post-stroke (39, 40). These alterations may have made the structure of the FMA-UE more variant.

“Hook grasp” was identified as a misfit in the present study, and this item was also erratic in the participants at 6 months post-stroke (22). The FMA-UE items were generated based on Brunnstrom motor testing. Brunnstrom (41) described the hook grasp as “holding onto the handles of a handbag placed in the hand,” which can be performed within flexor synergies, whereas Fugl-Meyer (12) defined this movement as “extending the metacarpophalangeal joints of digits II-V and flexing the proximal and distal interphalangeal joints,” which requires extensors and flexors individually. This modification possibly made the task different from the originally intended movement for the paretic hand, thus making the item deviate from the construct measured by the other items. However, it remains to be controversial whether the hook grasp reflects motor function of a paretic upper extremity, because no motor control theory has been provided to support this, as noted in a previous study (22).

### Item Difficulty Hierarchy

In addition to the three reflex items that were also removed in the study by Woodbury et al. (21) “elbow flexion” and “finger mass flexion” were omitted in the present study. Almost all participants obtained the highest score possible for these items in the present study, which suggests that the reflex and synergistic movements were the easiest items. The present results are consistent with the stepwise recovery course proposed in Fugl-Meyer's original article (12). However, the studies by Woodbury et al. showed that the item difficulty order did not follow the expected stepwise sequence, with synergies and each joint movement spanning the difficulty hierarchy (21, 22). As Woodbury et al. noted, the item difficulty hierarchy in persons with acute stroke with mild to moderate deficits would be arranged according to inherent task-specific demands of the movements (21). In contrast, the present findings suggest that the item difficulty in persons with chronic stroke with moderate to severe deficits would reflect synergies, as Fugl-Meyer had originally described (12). The difficulty hierarchy of grasp in the present study showed that gross movements using a whole hand (e.g., cylindrical and spherical) were easy, whereas coordinated movements using digits (e.g., pincer and thumb) were difficult, and these results were consistent with those of the previous studies, regardless of stroke chronicity (21, 42). The difficulty of finger mass extension occurred between cylindrical and spherical grasp in the present study. These findings suggest that the greater the space for grasping an object, the more difficult the movement will be, because it requires the ability to extend the fingers.

Finally, the movements that are difficult for persons with moderate to severe chronic upper limb paresis were identified. Although a few previous studies reported key movements predicting motor recovery or response to interventions in persons with severe chronic stroke (43, 44), limited research has been conducted with the aim of understanding how difficult they were compared to other movements. The present study filled the gap among the previous studies and enables comparison of each movement of paretic upper limbs using a measure of motion difficulty. For example, “shoulder flexion to 90° with elbow extended” was reported as a key movement (43). This item was among the easiest items next to synergies in the present study, and it might be a candidate initial target for stroke rehabilitation and for technology-aided/robotic therapy. We do not assume that this is an effective approach, which would require further investigations in clinical trials. Thus, the present findings provide an important piece of basic knowledge for rehabilitation targeted at persons with moderate to severe chronic stroke. This knowledge might help in selecting treatment targets, in which case using the 27-item FMA-UE might be beneficial. Creation of keyform recovery maps for persons with moderate to severe chronic stroke from these results would also be possible, but it requires further investigation in a population with a wider range of severity to ensure how far the difficulty hierarchy is maintained beyond the different upper limb functions.

The present study had several limitations. First, the cut-off values of severity for the FMA-UE were not established, and various values have been reported (45, 46). This study was designed around neurorehabilitation for persons with moderate to severe chronic stroke, so the cut-off values in a recent review of this field were used, and caution should be taken when using these results. MAS was also used to assess resistance to passive movement and future work could include a more specific measure of spasticity, such as the Tardieu scale, to account for this outcome (47). Second, although the FMA-UE includes multi-joint movements, not all combinations are assessed. The motion difficulty would change according to the positions of proximal joints, so care should be taken when interpreting the present results. Third, the participants were recruited in a single center, and the patient characteristics according to the institution affect the generalization of the results. For example, persons with apparent contracture on the paretic upper limb were excluded, so clinicians should be cautious when applying these results to persons with severe contractures, although this population is generally not likely to be eligible for upper limb motor rehabilitation. In addition, the item difficulty that matched the ability of the participants in this sample was estimated fairly accurately, but items that were too easy or too difficult for these participants were less accurately estimated; thus, the results of the present study cannot be applied to persons with the most severe or mildest deficits.

## Conclusions

The FMA-UE is a valid assessment tool of upper extremity motor function in persons with chronic stroke with moderate to severe deficits. The present results showed that item difficulty was consistent with the stepwise recovery course proposed by Fugl-Meyer. The upper extremity movements that are difficult for patients with moderate to severe chronic paresis were determined, which would enable comparison of each movement using a measure of motion difficulty in future studies.

## Data Availability Statement

The data analyzed in this study is subject to the following licenses/restrictions: Anonymized data sets are stored by researchers in accordance with ethical committee approval requirements. Requests to access these datasets should be directed to Michiyuki Kawakami, michiyukikawakami@hotmail.com.

## Ethics Statement

This study was approved by the institutional ethics review board (20190144). The outline of the study was published on the public website, and the participants were guaranteed the right to refuse participation.

## Author Contributions

NH and MK contributed to the study concept and design, data acquisition and analysis, data interpretation, and drafting of the manuscript. RI contributed to the study design, data acquisition and analysis, data interpretation, and drafting of the manuscript. KT and TN contributed to the data interpretation and data acquisition. KO and ML contributed to the data interpretation and editing of the manuscript. All authors contributed to the article and approved the submitted version.

## Conflict of Interest

MK and ML are founding scientists of the startup company Connect Inc. that facilitates the social implementation of university research results. KO is employed by Connect Inc. The remaining authors declare that the research was conducted in the absence of any commercial or financial relationships that could be construed as a potential conflict of interest.

## References

[1] Coscia M Wessel MJ Chaudary U Millán JDR Micera S Guggisberg A Neurotechnology-aided interventions for upper limb motor rehabilitation in severe chronic stroke. Brain. (2019) 142:2182–97. 10.1093/brain/awz181 31257411 PMC6658861

[2] Page SJ Gater DR Bach-Y-Rita P. Reconsidering the motor recovery plateau in stroke rehabilitation. Arch Phys Med Rehabil. (2004) 85:1377–81. 10.1016/j.apmr.2003.12.031 15295770

[3] McCabe J Monkiewicz M Holcomb J Pundik S Daly JJ. Comparison of robotics, functional electrical stimulation, and motor learning methods for treatment of persistent upper extremity dysfunction after stroke: a randomized controlled trial. Arch Phys Med Rehabil. (2015) 96:981–90. 10.1016/j.apmr.2014.10.022 25461822

[4] Daly JJ McCabe JP Holcomb J Monkiewicz M Gansen J Pundik S. Long-dose intensive therapy is necessary for strong, clinically significant, upper limb functional gains and retained gains in severe/moderate chronic stroke. Neurorehabil Neural Repair. (2019) 33:523–37. 10.1177/1545968319846120 31131743 PMC6625035

[5] Barker RN Brauer SG Carson RG. Training of reaching in stroke survivors with severe and chronic upper limb paresis using a novel nonrobotic device: a randomized clinical trial. Stroke. (2008) 39:1800–7. 10.1161/STROKEAHA.107.498485 18403742

[6] Pollock A Farmer SE Brady MC Langhorne P Mead GE Mehrholz J. Interventions for improving upper limb function after stroke. Cochrane Database Syst. (2014) 11:CD010820. 10.1002/14651858.CD010820.pub2 25387001 PMC6469541

[7] Taub E Uswatte G Elbert T. New treatments in neurorehabilitation founded on basic research. Nat Rev Neurosci. (2002) 3:228. 10.1038/nrn754 11994754

[8] Timmermans AA Spooren AI Kingma H Seelen HA. Influence of task-oriented training content on skilled arm-hand performance in stroke: a systematic review. Neurorehabil Neural Repair. (2010) 24:858–70. 10.1177/1545968310368963 20921325

[9] Makino H Hwang EJ Hedrick NG Komiyama T. Circuit mechanisms of sensorimotor learning. Neuron. (2016) 92:705–21. 10.1016/j.neuron.2016.10.029 27883902 PMC5131723

[10] Plautz EJ Milliken GW Nudo RJ. Effects of repetitive motor training on movement representations in adult squirrel monkeys: role of use versus learning. Neurobiol Learn Mem. (2000) 74:27–55. 10.1006/nlme.1999.3934 10873519

[11] Rong W Li W Pang M Hu J Wei X Yang B. A neuromuscular electrical stimulation (NMES) and robot hybrid system for multi-joint coordinated upper limb rehabilitation after stroke. J Neuroeng Rehabil. (2017) 14:34. 10.1186/s12984-017-0245-y 28446181 PMC5406922

[12] Fugl-Meyer AR Jääskö L Leyman I Olsson S Steglind S. The post-stroke hemiplegic patient, 1. A method for evaluation of physical performance. Scand J Rehabil Med. (1975) 7:13–31. 1135616

[13] Baker K Cano SJ Playford ED. Outcome measurement in stroke: a scale selection strategy. Stroke. (2011) 42:1787–94. 10.1161/STROKEAHA.110.608505 21566236

[14] Kwakkel G Lannin NA Borschmann K English C Ali M Churilov L. Standardized measurement of sensorimotor recovery in stroke trials: consensus-based core recommendations from the Stroke Recovery and rehabilitation roundtable. Int J Stroke. (2017) 12:451–61. 10.1177/1747493017711813 28697709

[15] Gladstone DJ Danells CJ Black SE. The fugl-meyer assessment of motor recovery after stroke: a critical review of its measurement properties. Neurorehabil Neural Repair. (2002) 16:232–40. 10.1177/154596802401105171 12234086

[16] Lin JH Hsu MJ Sheu CF Wu TS Lin RT Chen CH. Psychometric comparisons of 4 measures for assessing upper-extremity function in people with stroke. Phys Ther. (2009) 89:840–50. 10.2522/ptj.20080285 19556333

[17] Platz T Pinkowski C van Wijck F Kim IH di Bella P Johnson G. Reliability and validity of arm function assessment with standardized guidelines for the Fugl-meyer test, action research arm test and box and block test: a multicentre study. Clin Rehabil. (2005) 19:404–11. 10.1191/0269215505cr832oa 15929509

[18] Sullivan KJ Tilson JK Cen SY Rose DK Hershberg J Correa A. Fugl-Meyer assessment of sensorimotor function after stroke: standardized training procedure for clinical practice and clinical trials. Stroke. (2011) 42:427–32. 10.1161/STROKEAHA.110.592766 21164120

[19] See J Dodakian L Chou C Chan V McKenzie A Reinkensmeyer DJ. A standardized approach to the Fugl-Meyer assessment and its implications for clinical trials. Neurorehabil Neural Repair. (2013) 27:732–41. 10.1177/1545968313491000 23774125

[20] Amano S Umeji A Uchita A Hashimoto Y Takebayashi T Takahashi K. Clinimetric properties of the Fugl-Meyer assessment with adapted guidelines for the assessment of arm function in hemiparetic patients after stroke. Top Stroke Rehabil. (2018) 25:500–8. 10.1080/10749357.2018.1484987 30028660

[21] Woodbury ML Velozo CA Richards LG Duncan PW Studenski S Lai SM. Dimensionality and construct validity of the fugl-meyer assessment of the upper extremity. Arch Phys Med Rehabil. (2007) 88:715–23. 10.1016/j.apmr.2007.02.036 17532892

[22] Woodbury ML Velozo CA Richards LG Duncan PW Studenski S Lai SM. Longitudinal stability of the fugl-meyer assessment of the upper extremity. Arch Phys Med Rehabil. (2008) 89:1563–9. 10.1016/j.apmr.2007.12.041 18674991

[23] Fujiwara T Kasashima Y Honaga K Muraoka Y Tsuji T Osu R. Motor improvement and corticospinal modulation induced by hybrid assistive neuromuscular dynamic stimulation (HANDS) therapy in patients with chronic stroke. Neurorehabil Neural Repair. (2009) 23:125–32. 10.1177/1545968308321777 19060131

[24] Okuyama K Ogura M Kawakami M Tsujimoto K Okada K Miwa K. Effect of the combination of motor imagery and electrical stimulation on upper extremity motor function in patients with chronic stroke: preliminary results. Ther Adv Neurol Disord. (2018) 11:1–10. 10.1177/1756286418804785 30327684 PMC6178123

[25] Mizuno K Abe T Ushiba J Kawakami M Ohwa T Hagimura K. Evaluating the effectiveness and safety of the electroencephalogram-based brain-machine interface rehabilitation system for patients with severe hemiparetic stroke: protocol for a randomized controlled trial (BEST-BRAIN Trial). JMIR Res Protoc. (2018) 7:e12339. 10.2196/12339 30522993 PMC6302229

[26] University Hospital Medical Information Network UMIN-Clinical Trial Registry. Available online at: https://upload.umin.ac.jp/cgi-open-bin/ctr_e/ctr_view.cgi?recptno=R000028109 (accessed June 30, 2020)

[27] Platz T. ARM, Arm Rehabilitation Measurement: Manual for Performance and Scoring of the Fugl-Meyer Test (Arm Section), Action Research Arm Test, and the Box-and-Block Test. Baden-Baden: Deutscher Wissenschafts-Verlag (2005).

[28] Chino N Sonoda S Domen K Saitoh E Kimura A Stroke impairment assessment set (SIAS). In: Chino N, Melvin JL, editors. Functional Evaluation of Stroke Patients. Tokyo: Springer-Verlag (1995). p. 19–31. 10.1007/978-4-431-68461-9_3

[29] Bohannon RW Smith MB. Interrater reliability of a modified ashworth scale of muscle spasticity. Phys Ther. (1987) 67:206–7. 10.1093/ptj/67.2.206 3809245

[30] Hong I Bonilha HS. Psychometric properties of upper extremity outcome measures validated by rasch analysis: a systematic review. Int J Rehabil Res. (2017) 40:1–10. 10.1097/MRR.0000000000000202 27755166

[31] Streiner DL Norman GR Cairney J Health Measurement Scales: A Practical Guide to Their Development and Use. Oxford: Oxford University Press (2015). 10.1093/med/9780199685219.003.0001

[32] Andrich D A rating formulation for ordered response categories. Psychometrika. (1978) 43:561–73. 10.1007/BF02293814

[33] Woodbury ML Anderson K Finetto C Fortune A Dellenbach B Grattan E. Matching task difficulty to patient ability during task practice improves upper extremity motor skill after stroke: a proof-of-concept study. Arch Phys Med Rehabil. (2016) 97:1863–71. 10.1016/j.apmr.2016.03.022 27117385 PMC5278766

[34] Brainin M Norrving B Sunnerhagen KS Goldstein LB Cramer SC Donnan GA. Poststroke chronic disease management: towards improved identification and interventions for poststroke spasticity-related complications. Int J Stroke. (2011) 6:42–6. 10.1111/j.1747-4949.2010.00539.x 21205240

[35] Gracies JM. Pathophysiology of Spastic Paresis. I: paresis and soft tissue changes. Muscle Nerve. (2005) 31:535–1. 10.1002/mus.20284 15714510

[36] McMorland AJ Runnalls KD Byblow WD. A neuroanatomical framework for upper limb synergies after stroke. Front Hum Neurosci. (2015) 9:82. 10.3389/fnhum.2015.00082 25762917 PMC4329797

[37] Urban PP Wolf T Uebele M Marx JJ Vogt T Stoeter P. Occurrence and clinical predictors of spasticity after ischemic stroke. Stroke. (2010) 41:2016–20. 10.1161/STROKEAHA.110.581991 20705930

[38] Wissel J Manack A Brainin M. Toward an epidemiology of poststroke spasticity. Neurology. (2013) 80:S13–9. 10.1212/WNL.0b013e3182762448 23319481

[39] Roh J Rymer WZ Perreault EJ Yoo SB Beer RF. Alterations in upper limb muscle synergy structure in chronic stroke survivors. J Neurophysiol. (2013) 109:768–81. 10.1152/jn.00670.2012 23155178 PMC3567389

[40] Cirstea MC Levin MF. Compensatory strategies for reaching in stroke. Brain. (2000) 123:940–53. 10.1093/brain/123.5.940 10775539

[41] Brunnstrom S. Motor testing procedures in hemiplegia: based on sequential recovery stages. Phys Ther. (1966) 46:357–75. 10.1093/ptj/46.4.357 5907254

[42] Persch AC Gugiu PC Velozo CA Page SJ. Rasch analysis of the wrist and hand fugl-meyer: dimensionality and item-level characteristics. J Neurol Phys Ther. (2015) 39:185–92. 10.1097/NPT.0000000000000096 26050075 PMC4470757

[43] McPherson JG Chen A Ellis MD Yao J Heckman CJ Dewald JPA. Progressive recruitment of contralesional cortico-reticulospinal pathways drives motor impairment post stroke. J Physiol. (2018) 596:1211–25. 10.1113/JP274968 29457651 PMC5878212

[44] Lang CE Wagner JM Edwards DF Sahrmann SA Dromerick AW Lang CE. Recovery of grasp versus reach in people with hemiparesis poststroke. Neurorehabil Neural Repair. (2006) 20:444–54. 10.1177/1545968306289299 17082499

[45] Woodbury ML Velozo CA Richards LG Duncan PW. Rasch analysis staging methodology to classify upper extremity movement impairment after stroke. Arch Phys Med Rehabil. (2013) 94:1527–33. 10.1016/j.apmr.2013.03.007 23529144

[46] Dobkin BH Carmichael ST. The specific requirements of neural repair trials for stroke. Neurorehabil Neural Repair. (2016) 30:470–8. 10.1177/1545968315604400 26359342 PMC4786476

[47] Sunnerhagen KS Olver J Francisco GE. Assessing and treating functional impairment in poststroke spasticity. Neurology. (2013) 80:S35–44. 10.1212/WNL.0b013e3182764aa2 23319484

