# Supplementary material for: Item Difficulty of Fugl-Meyer Assessment for Upper Extremity in Persons With Chronic Stroke With Moderate-to-Severe Upper Limb Impairment
Source: Front Neurol. 2020 Nov 16;11:577855. doi: 10.3389/fneur.2020.577855 (PMC7701100; doi:10.3389/fneur.2020.577855)
Supplement: Supplementary file 1 [file Table_1.docx]

Supplementary Material

# Supplementary Table 1. Outlines of the prior clinical trials

|  | Study design | Inclusion criteria | Exclusion criteria | Intervention | Outcome measures | ethical approval/ clinical trial registration numbers |
| --- | --- | --- | --- | --- | --- | --- |
| Hybrid assistive neuromuscular dynamic stimulation therapy | pre–post test, cohort design | (1) time from stroke onset >150 days; (2) no cognitive deficits;(3) no pain in the paretic UE; (4) pROM >0 degrees of the affected wrist and −10 degrees of MCP joints; (5) detectable surface EMG signals in the affected EDC; (6) no severe proprioceptive deficit in the affected UE; (7) the ability to walk without physical assistance; (8) no motor improvement in the last 1 month before the intervention. | (1) history of major psychiatric or previous neurological diseases, including seizures; (2) cognitive impairment precluding appropriately giving informed consent or MMSE <25/30; (3) use of drugs active in the CNS. | facilitate the use of the hemiparetic UE in daily life by combining closed-loop EMG-controlled NEMS with a wrist-hand splint | FMA-UE; SIAS; MAL; MAS | Institutional Ethics Review Board of Keio University School of Medicine (20180174) |
|  |  |  |  |  |  |  |
| Combination of motor imagery and electrical stimulation | single group, pre- and post-intervention design. | (1) a first unilateral stroke; (2) time from stroke onset >150 days; (3) inability to extend the paretic fingers; (4) pROM >−30 degrees for MCP joint extension; (4) no pacemaker or other implanted stimulator. | (1) serious medical conditions; (2) pacemaker or use of other implanted stimulators; (3) history of seizures within 90 days before enrollment. | The combination of motor imagery (MI) and afferent input with electrical stimulation (ES) applied to the radial nerve at the spiral groove | FMA-UE; MAL; MAS | Institutional Ethics Review Board (20170253)/ UMIN Clinical Trial Registry (UMIN000023731) |
|  |  |  |  |  |  |  |
| Electroencephalogram-based brain-machine interface rehabilitation | single-blinded, multicenter RCT using a parallel arm design | (1) time from stroke onset >90 days; (2) first ever stroke patients with UE paresis; (3) no loss of proprioception in paretic fingers; (4) ability to raise the paretic hand to the height of the nipple; (5) pROM >−10 degrees for MCP joint extension; (6) ability to flex the paretic fingers voluntarily but not to extend them; (7) ability to walk independently with or without assistance; (8) ability to understand and consent to the study protocol; (9) aged ≥18 years. | (1) serious medical conditions; (2) pacemaker or use of other implanted stimulators; (3) history of seizures within 90 days before enrollment; (4) participation in another clinical trial for regulatory approval within 90 days before enrollment; (5) receiving other special neurorehabilitation techniques for UE paresis within 90 days before enrollment; (6) injection of botulinum toxin or phenol for treatment of UE spasticity within 90 days before enrollment; (7) impossible to record EEG because of skin status or skull deformity | brain-machine interface to detect event-related desynchronization on scalp EEG and to operate a motor-driven hand orthosis combined with neuromuscular electrical stimulation | FMA-UE; ARAT; MAL; SIAS; MAL | Institutional Review Board of Keio University (protocol number: KCTR-D008, reference number: D16-03)/ UMIN Clinical Trials Registry (UMIN000026372)/ International Registered Report Identifier (IRRID): DERR1-10.2196/12339 |
|  |  |  |  |  |  |  |
| Modified constraint induced movement therapy | single group, pre- and post-intervention design. | chronic stroke patients with hemiplegia >6month from the onset; mild cognitive dysfunction and/or aphasia is permitted with MMSE >20/30 and to be able to cooperate with the intervention; independent in ambulatory and basic ADL;SIAS proximal UE ≥3, distal UE ≥1b; No contracture or severe pain; aged≥12 years | contraindication for physical therapies | Limitation of use of non-paretic hand using keeper glove in daily life scene | FMA-UE; SIAS; WMFT; MAL | Institutional Ethics Review Board (20160323)/ UMIN Clinical Trial Registry (UMIN000024425) |
|  |  |  |  |  |  |  |

Abbreviations: UE, upper extremity; pROM, passive range of motion; MCP, metacarpopharyngeal; EMG, electromyography; EDC, extensor digitorum communis; MMSE, Mini-Mental State Examination; CNS, central nervous system; NMES, neuromuscular electrical stimulation; FMA-UE, Fugl-Meyer Assessment for Upper Extremity; SIAS, Stroke Impairment Assessment Set; MAL, Motor Activity Log; MAS, modified Ashworth scale; EEG, electroencephalography; ARAT, Action Research Arm Test; ADL, activities of daily living; WMFT, Wolf Motor Function Test.
